# An Alternative Viewpoint—Comment on Prescott and Bland *Int. J. Environ. Res. Public Health* 2020, *17*, 1407

**DOI:** 10.3390/ijerph17145004

**Published:** 2020-07-11

**Authors:** Roy D. Sleator

**Affiliations:** Department of Biological Sciences, Munster Technological University, Bishopstown Campus, T12 P928 Cork, Ireland; roy.sleator@cit.ie; Tel.: +353-21-433-5405

I would like to commend Prescott and Bland [1] on their entertaining *Viewpoint* on Dubos’ Spaceship Earth. However, I am disappointed by their assertion that space science is “*a form of intellectual escapism*” and worse, a distraction from “*more pressing priorities here on Earth*”.

Even more disheartening is the negative view of the “*enthusiasm and hyperbole reminiscent of the 1960s*” which they attach to our current and future view of space [2]. I would argue that without the enthusiasm of the 1960s we might not have put a man on the moon [3], robots on Mars [4] or sent Voyager 1 on its ultimate path to interstellar space [5]. All of this, lest we forget, was achieved in a period of our history during which there were far “*more pressing priorities here on Earth*”. Indeed, 1960s Earth, under the shadow of the cold war and nuclear arms proliferation, was arguably a far less stable place than it is today [6]. Yet, against this backdrop, John F. Kennedy roused the nations of the world in 1962 with a defiant statement of intent: “*We choose to go to the moon in this decade and do the other things. Not because they are easy, but because they are hard*”.

With this in mind, I would like to posit an alternative viewpoint to that of Prescott and Bland [1], one which resonates with Stephen Hawking’s advice that we, the citizens of Earth, “*remember to look up at the stars and not down at your feet.…it matters that you don’t just give up*.”

Indeed, looking to the stars does not necessarily imply that we have given up on Earth. On the contrary, two of our most high-profile space entrepreneurs, Jeff Bezos and Elon Musk, have pledged significant resources to solving some of the most “*pressing priorities here on Earth*”, while also reaching for the stars. Bezos, the founder of Blue Origin, a company whose mission is “*building a road to space so our children can build the future*”, has recently pledged USD 10 billion of his personal fortune to tackle climate change [7]. While Elon Musk, founder of SpaceX, a company “*with the ultimate goal of enabling people to live on other planets*”, is also the CEO of Tesla, a company which has revolutionized the automotive industry, dramatically reducing our dependence on the internal combustion engine and fossil fuels [8]. Furthermore, while I agree with Prescott and Bland [1] that “*tremendous resources are directed at planning for crewed flights and colonization on Mars*”, these resources are, for the most part, privately funded (at least in the case of Blue Origin and SpaceX), and as such pose little direct financial burden on the public purse.

Finally, I will conclude by echoing Musk’s sentiments—“*If the future does not include being out there among the stars and being a multi-planet species, I find that incredibly depressing*”.
